# Creating effective career development programs

**DOI:** 10.1017/cts.2016.30

**Published:** 2017-04-18

**Authors:** Doris McGartland Rubio, Georgeanna F. W. B. Robinson, Janice Gabrilove, Emma A. Meagher

**Affiliations:** 1 Department of Medicine, Division of General Internal Medicine, University of Pittsburgh School of Medicine, Pittsburgh, PA, USA; 2 Institute for Clinical Research Education, University of Pittsburgh School of Medicine, Pittsburgh, PA, USA; 3 Clinical and Translational Science Institute, University of Pittsburgh, Pittsburgh, PA, USA; 4 Qualitative Research, Analytic Support and Institutional Research, Grinnell College, Grinnell, IA, USA; 5 Tisch Cancer Institute, Icahn School of Medicine at Mount Sinai, New York, NY, USA; 6 ConduITS, The Institutes for Translational Sciences, Icahn School of Medicine, New York, NY, USA; 7 Clinical Research Education Program, Graduate School of Biomedical Sciences, Icahn School of Medicine at Mount Sinai, New York, NY, USA; 8 Perelman School of Medicine, The Institute of Translational Medicine and Therapeutics, University of Pennsylvania, Philadelphia, PA, USA

**Keywords:** Education, Training, Clinical and translational science workforce, Clinical and translational science awards

## Abstract

This paper is the fourth in a 5-part series that focuses on educating and training the clinical and translational science workforce. The goal of this paper is to delineate components of effective career development programs that go beyond didactic training. All academic health centers with a Clinical and Translational Science Award have a KL2 career development award for junior faculty, and many also have a TL1 training program for predoctoral and postdoctoral fellows. The training across these programs varies, however junior investigators across the United States experience similar challenges. Junior investigators can get overwhelmed with the demands of building their own research program, particularly in academia. 1Often, they are sidetracked by competing demands that can derail their progress. In these situations, junior investigators experience frustration and may search for alternative career paths. By providing them with additional professional skills in the 5 domains of: (1) self-awareness; (2) selecting the right topic and securing funding; (3) getting adequate support; (4) working with others; and (5) managing yourself, your career, and your demands. We will give junior investigators additional tools to manage these demands and facilitate their own career success.

## Introduction

This paper is the fourth in a 5-part series on the clinical and translational science educational pipeline [1]. The overall goal of this series is to describe how institutions can develop an effective educational pipeline along the entire academic and career development continuum. Here, we focus on individual skills that career programs can provide to trainees to accelerate their career success.

The University of Pittsburgh’s Research on Careers (ROC) workgroup developed a model of career success [2]. In this model, career success has 2 dimensions—extrinsic (eg, grants) and intrinsic (eg, career satisfaction)—and 2 main contributing factors—personal and organizational. Within these components are several modifiable elements, which can increase trainees’ success, such as training, leadership, and mentoring.

A workgroup from the Clinical and Translational Science Award (CTSA) education and training arm conducted a qualitative study on the basis of ROC’s model to examine factors that contribute to successful transition to independence [3]. Forty former KL2 or K12 scholars (20 independently funded and 20 not independently funded) from multiple institutions were interviewed. The results of the study support ROC’s theoretical model and found additional factors that could be added. The personal factors identified by the participants included networking, resilience, initiative in career development, autonomy over work, and ability to balance work and personal demands. The organizational factors that impacted career success, as noted by the scholars, included mentoring, protected time for research, and resources. This work further clarified and underscored the need for training that goes beyond the traditional academic, discipline-based curriculum for junior investigators.

Many of the personal factors and academic life skills that influence career success are trainable. Skills such as resilience and initiative can be taught, as can leadership and time management. Our previous work has shown that these factors are needed for career success; however, these critically important components are typically not included in the context of didactic clinical translational science degree programs. Many of these programs generally provide research skill training that is limited to methodology and statistics, which falls short of skills required for career success. In addition, mentors usually do not focus on helping their mentees develop the necessary personal factors for success, which can leave trainees alone to figure out work-life integration or how to negotiate for resources needed for their research. Many early-stage trainees are not successful in managing this critical aspect of their careers and consequently leave academic research for alternative career paths. Effective career development programs that include a focus on professional skill development could greatly assist these trainees in ensuring successful research career outcomes.

## Components of Career Development Programs

Table 1 outlines critical components of a career development program that are beneficial for junior investigators as they develop their career in clinical and translational research. These needs fall into 5 broad categories: (1) self-awareness; (2) selecting the right topic and securing funding; (3) getting adequate support; (4) working with others; and (5) managing yourself, your career, and your demands. Individual components of these specific educational domains have been included in various training initiatives; however, career development programs in translational science rarely offer all of these essential constituents as an integrated effort designed to best assure the success of junior investigators.

**Table 1:** Critical components of career development programs

Categories	Educational needs
Self-awareness	Personality “job fit” assessment
	360° evaluations
	Working and communication style
	Resilience
	Initiative
Selecting the right topic and securing funding	Critical thinking and problem solving abilities
	Asking the right question
	“Outside the box” thinking
	Grant writing
	Medical writing
Getting adequate support	Building and working with an effective mentoring team
	Negotiation skills
	Securing needed resources including protected time
Working with others	Oral communication skills
	Effective peer reviewing
	Group dynamics and multidisciplinary teams
	Networking skills
Managing yourself, your career, and your demands	Leadership
	Career coaching
	Time management
	Work and life integration

### Self-Awareness

Many disciplines outside science and medicine have had considerable success embracing the use of validated tools to identify candidates who are a good match for a particular position in a specific work environment [4]. More recently, determination of surgical training applicants’ personal talents and behavioral styles has been undertaken with considerable success using the TriMetrix Personal Talent Report (Target Training International Ltd, Scottsdale, AZ, USA), with the goal of selecting more appropriate candidates for a specific surgical training program [5]. The Myers-Briggs Type Indicator has also been shown to delineate individual characteristics associated with choice of medical subspecialty and has been used in specific faculty development and leadership programs in an effort to enhance self-awareness/evaluation, thereby enabling individuals to identify preferences and optimally apply their respective talents more successfully [6, 7]. These same types of programs have also begun to incorporate resilience training. Similarly, 360° feedback has provided useful and impactful outcomes in the workplace and in medical training programs for a considerable period of time [8]. However, these tools have not been universally adapted for emerging clinical translational investigators to provide objective and constructive feedback needed for their development.

### Selecting the Right Topic and Securing Funding

We anticipate that junior investigators have broadly identified their area of research interest but are often challenged with moving these interests into a research program. Many junior investigators are never trained to critically review the relevant literature. Without this grounding in their field, it is difficult to arrive at novel and creative questions that are both answerable and fundable. An additional challenge is “finding the data” (eg, literature, publically available databases, local and national clinical data repositories, digital and internet resources). While some clinical and translational science degree programs have incorporated “finding the data” in their didactic courses, investigators who are not formally trained rarely get exposed to this in career development programs.

Intensive grant writing courses or workshops can also assist junior investigators in this particular domain for career success. Such courses work best when instructors are faculty members particularly skilled in facilitating idea development and reviewing grants. Meeting weekly, these courses can progress through each part of a typical grant, including specific aims, background, innovation, approach, limitations, and qualifications of investigative team. At the University of Pittsburgh, Icahn School of Medicine at Mount Sinai, and University of Pennsylvania, trainees work in small groups (no larger than 6–8 trainees and 1 instructor), organized broadly by type of research. For example, groups could be organized by T1 translational research, health services research, clinical trials, and so forth. Following some didactic training on each part of the grant, trainees work intensively between group sessions to write the relevant part of their own grant before submitting it to the group for peer feedback. Following feedback, trainees rewrite the section and resubmit. This iterative process gives them a chance to think critically about their proposed project and justify their approach. Trainees refine their questions until they are appropriately articulated and focused, and they hone their grantsmanship as well as their written communication skills.

For many junior investigators, instruction on medical writing can be beneficial. Trainees need to learn: how to find their own effective writing process; how to write in appropriate styles and adapt to one’s audience; the components of typical research reports; appropriate responses to reviewers; and expectations for peer reviewing. Practicing writing and receiving intensive, specific, and formative feedback about their own writing allows junior investigators to establish early effective writing habits, which ought to translate into productivity later in their careers.

### Getting Adequate Support

Junior investigators need a broad range of support to develop their research interests into funded research projects and ultimately published manuscripts. Once a junior investigator has begun to outline a research project, mentors with relevant expertise can be instrumental in helping the investigator answer his or her research questions. However, an effective mentoring team requires more than a collection of relevant content experts. Career development program leaders can facilitate the creation of mentoring teams by providing knowledge of both local and national colleagues who have a history of effective mentoring. Program leaders can make introductions where necessary, provide oversight to ensure that the full mentoring team meets regularly, and intervene to assist with challenges or conflicts that occur.

Mentee training can also be helpful for junior investigators who may not realize the extent to which they ought to steer and manage their team of mentors. Mentees need to learn to: develop agendas, follow up with individuals after meetings, keep track of questions and progress between meetings, keep their career and research goals at the center of the team’s efforts, and manage their mentors’ different mentoring styles. They may also need assistance in accepting contradictory feedback from multiple mentors and deciding on their own approach toward mentors who cannot agree on the best course of action.

Mentors, particularly faculty with many years of experience, may have established their mentoring techniques and styles and be reticent to develop mentoring skills further or experiment with new techniques. However, given the multiple challenges to effective mentoring, senior mentors can benefit from mentor training. A mentor training program, such as the one found to be effective by investigators at the University of Wisconsin-Madison [9], can introduce mentors to alternative ways to approach common mentoring problems, provide new perspectives and concepts for consideration, offer alternative opinions and courses of actions suggested by peers, and serve as a safe and confidential space to discuss current mentoring challenges. Being mindful of mentoring skills allows mentors to be intentional about specific aspects of mentoring that have been shown to help but that may have been given less attention by mentors previously, such as opening a mentor’s network to the mentee, adapting mentoring style to each mentee, respecting each mentee’s individual career goals, and providing resources, where possible, to support mentees.

Junior investigators need support from their department in concert with their mentors. Too often, junior faculty members are asked to serve on time-demanding committees or to pick up additional clinical time, such as urgent care. Department chairs need to protect junior investigators from these commitments; it can be difficult for someone who is junior to deny the request of a more senior person. If the department chair does not ensure that the junior investigator’s research time is protected, and that undue demands are not imposed, then it will be impossible for that junior faculty member to be an investigator. One cannot build a research career on nights and weekends.

### Working with Others

Junior investigators need to be able to function as part of multidisciplinary teams, as well as to be able to lead teams in pursuit of their own research agenda. Overcoming multiple disciplinary differences is critical, which may include the customs and expectations of working together, the language of research, epistemologies, and methodologies. Career development programs can train investigators to work patiently through problems until they arrive at a mutual understanding, and to present issues in a clear manner that is free of disciplinary jargon.

Many of the skills that are instrumental for highly functioning multidisciplinary teams are also helpful when leading and managing one’s own research team. Business schools have long taught leadership and management skills, but this expertise does not consistently parlay into other parts of universities where clinical and translational investigators may be working. A formal, didactic program can be followed to ensure a thorough tutelage in the key aspects of leadership and management. If time and/or tuition costs present a barrier to this approach, however, workshops can also provide some useful training to investigators. Topics that are particularly relevant include strategic planning of one’s research agenda, motivating others, effective listening, communication skills, managing conflict, personnel management, and budgeting. In workshops or courses, investigators may find it helpful to discuss cases in small groups, complete an assignment that applies something to their own research group, and then report back to the group or class; this process results in an iterative approach to trying out and refining skills.

The acquisition of networking skills is critical to the process of working with others in an optimal way, and also serves to expand further opportunities for mentorship in complementary areas of relevance to one’s ongoing research. KL2 alumni highlighted networking as a critically important skill associated with, or impeding, successful transition to independence [3]. Formal curricular components and/or workshops focusing on the development of this informative ability are sorely needed as a key component of career development programs.

### Managing Yourself, Your Career, and Your Demands

Junior investigators commonly experience overwhelming demands on their time, both personally and professionally. It is critical that they learn early in a career to manage their time and discover how they work most efficiently and effectively. Career development programs can offer workshops with productivity tips, suggestions to aid efficiency, and ideas to prompt junior investigators to develop habits that will allow them to manage their time better. However, it may be more helpful if mentors make suggestions and intentionally role model their time management techniques. Mentors can shield mentees from burdensome service duties at this point in their careers and help mentees prioritize work appropriately.

**Fig. 1:**
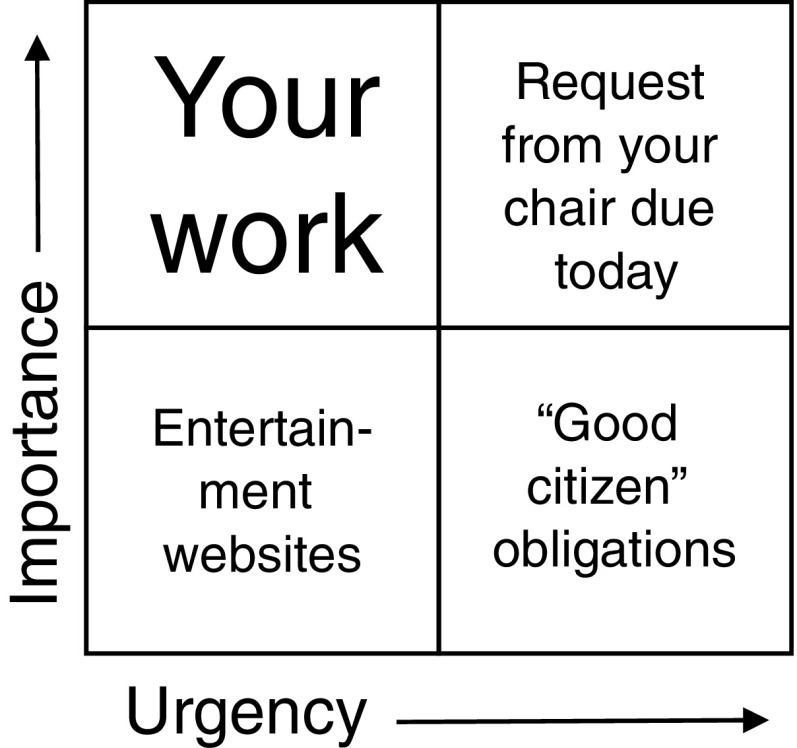
Prioritization of important work over urgent work.

Particularly helpful is the 2×2 table that distinguishes urgent from important work (Fig. 1). Junior investigators often find that they spend significant amounts of time on tasks that contribute little toward their main career goal. As part of a department or campus community, there are tasks that must be completed to make an individual faculty member a “good citizen” on their campus. However, those who complete such tasks well and without complaint, frequently find themselves unduly burdened by more such work, preventing them from focusing on their research. These tasks fall into the high-urgency/low-importance (to one’s career) category because they frequently have deadlines but contribute little to career progress. Other tasks, such as requests from a department chair or a funding agency need to be completed immediately. These are highly urgent and highly important to one’s career and must be prioritized. Unfortunately, there are frequently few deadlines in research, making one’s own work low on the urgency scale but undeniably high on the importance scale. Junior investigators need permission and encouragement from mentors, program directors, and administrators to put their own needs first and manage their time successfully.

The second part training investigators to manage themselves is to help them learn their own working style. Training programs could use standardized tests that offer some insight into personal styles such as the Myers-Briggs Type Indicator. These tests can also provide trainees with networks of similar scholars so they can develop relatedness with colleagues. By understanding their own strengths and weaknesses, tendencies, areas of difficulty, and effective habits that are practiced without thought, trainees can more effectively manage themselves. For example, if a trainee understands that he or she is more productive around 5 am, then it is important to organize the day to allow for an early awakening and some work before the hectic matters of the day evolve. Also, once investigators understand how they communicate, they can better facilitate effective communication and go some way to maintaining collegial relations.

Finally, developing effective time management skills can significantly benefit an investigator throughout his or her career. There are several effective books that teach time management skills, such as *Getting Things Done: The Art of Stress-Free Productivity* [10]. For example, although a common practice, time management experts agree you should not start your day with e-mail [11]. Instead, do the hardest work of the day, such as writing a section of your grant. Doing the hardest work before the brain becomes cluttered with urgent and unimportant issues facilitates superior work and leads to better productivity. Junior investigators need to learn to block time to write and to keep that time sacred, create internal deadlines and share these with mentors so that they cannot easily be dropped, and delegate tasks when appropriate.

## CTSA Career Development Programs: Lessons Learned

The CTSA program has been in existence since 2006. As part of the CTSA infrastructure, each CTSA institution has a career development award for junior faculty, funded under the KL2 mechanism. The overall goal of the KL2 is to develop independent investigators, but there are variabilities across institutions. The number of scholars supported by the KL2 mechanism varies; some programs are small with 2 scholars, while others may support 20 scholars. Scholars are supported for a minimum of 2 years and a maximum of 5 years. Robinson *et al*. [3] reported that scholars supported for only 2 years felt that this was insufficient time in which to secure their own funding—either another K award or an R01. Scholars supported for a longer length of time felt that they were better positioned to be productive and launch their independent careers. Some KL2 programs encourage scholars to earn an MS or certificate degree, whereas others have less structure to their training. The most important factor is that scholars can secure the training that they need to pursue their research agenda successfully. Although the training varies between programs, 1 commonality is that most program directors meet regularly with their scholars to ensure that scholars’ research is progressing as planned.

Another component of the CTSA program is the predoctoral and postdoctoral training grant, funded under a TL1 mechanism. Like the KL2 programs, the training that comprises different TL1 programs varies in size, intensity, and expectations. Some programs focus more on methods and scientific content; others also provide a range of professional skills, experiences, and training opportunities. TL1 fellows are expected to attend national meetings and present on their research as part of their academic socialization and to enhance their networks.

Beyond these formal training awards that provide financial support, most, if not all, CTSAs have other career development programs. These programs provide specific training for a range of trainees and on a range of topics (eg, training in mentoring; K to R programs; programs for investigators from groups under-represented in research; programs for medical students, residents, and basic researchers moving into more translational and clinical research). The range, breadth, and depth fluctuate across CTSAs. Some programs may have competitive admission and a year-long attendance requirement. Others may involve short modules, 1-hour workshop, or online videos for self-study.

There appear to be a limited number of programs that offer some of the personal factors that have been found to facilitate success, such as those discussed above. Curricular integration of these identified key elements into an innovative and unified platform would impact the success of future emerging clinical translational scientists in meaningful ways.

## Future Needs to Develop the Workforce

With the average age of research independence steadily increasing [12], the United States is in need of effective training programs that provide new investigators with critical skills needed to be successful in research. We need to help trainees with resilience and persistence so that they do not choose alternate career paths, but rather stay engaged in research. By including training on professional skills such as those described above, we can help these trainees successfully navigate their research careers and ultimately build a successful research program. As we develop best practices in training and career development, it is critical that we evaluate our efforts so that we can widely disseminate novel approaches.
